# Non-invasive Imaging in Women With Heart Failure — Diagnosis and Insights Into Disease Mechanisms

**DOI:** 10.1007/s11897-022-00545-2

**Published:** 2022-05-04

**Authors:** Rebecca Kozor, Aderonke Abiodun, Katharine Kott, Charlotte Manisty

**Affiliations:** 1 Faculty of Medicine and Health, University of Sydney, Sydney, Australia; 2 Royal North Shore Hospital, Sydney, Australia; 3 Institute of Cardiovascular Science, University College London, London, UK; 4 Barts Heart Centre, St Bartholomew’s Hospital, West Smithfield, London, UK

**Keywords:** Imaging, Cardiovascular magnetic resonance, Echocardiography, Imaging, Heart failure, Women

## Abstract

**Purpose of Review:**

To summarise the role of different imaging techniques for diagnosis and investigation of heart failure in women.

**Recent Findings:**

Although sex differences in heart failure are well recognised, and the scope of imaging techniques is expanding, there are currently no specific guidelines for imaging of heart failure in women.

**Summary:**

Diagnosis and stratification of heart failure is generally performed first line using transthoracic echocardiography. Understanding the aetiology of heart failure is central to ongoing management, and with non-ischaemic causes more common in women, a multimodality approach is generally required using advanced imaging techniques including cardiovascular magnetic resonance imaging, nuclear imaging techniques, and cardiac computed tomography. There are specific considerations for imaging in women including radiation risks and challenges during pregnancy, highlighting the clear unmet need for cardiology and imaging societies to provide imaging guidelines specifically for women with heart failure.

## Introduction

It is increasingly recognised that there are sex differences in the epidemiology, aetiology, presentation, and outcomes of heart failure [1, 2], and that these should be reflected in patient management. Position statements from leading cardiovascular organisations now include recommendations specific for women [3•]; however, dedicated guidelines specific to cardiac imaging in heart failure are lacking. Imaging is required throughout the clinical pathway of patients with heart failure, from confirming diagnosis to understanding aetiology, monitoring disease progression and response to treatment and risk stratification, and generally there is no universal strategy that will be optimal for all patients.

This review paper aims to summarise the role of cardiac imaging in women with heart failure, and identify the best imaging tools to address specific clinical scenarios in order to optimise clinical outcomes.

### Heart Failure in Women

Heart failure (HF) is an important cause of morbidity and mortality in women and it is estimated that 1 in 5 women will develop HF over the age of 40 years [4]. Women tend to develop HF at an older age in comparison to men, and non-ischaemic causes such as hypertension and valvular heart disease are more common.

HF in women is more commonly classified as heart failure with preserved (HFpEF) or mildly reduced ejection fraction (left ventricular ejection fraction 41–49%) than when compared to men, where left ventricular ejection fraction (LVEF) is more commonly reduced (LVEF ≤40%) [5]. Large registry studies have demonstrated that all patients with HF have high rates of 5-year mortality and rehospitalisation [6], and despite differences in baseline characteristics (females have more hypertension, anaemia, and depression, and less coronary artery disease (CAD), hyperlipidaemia, atrial fibrillation, and tobacco use) [7, 8], both men and women with HF with reduced ejection fraction (HFrEF) or HFpEF have similar rates of in-hospital mortality during an admission for acute decompensated heart failure [7].

Additionally, hospital admission rates for HF have decreased over time in men, but increased in women [9]. A recent meta-analysis of HF clinical trial data found that women with HFpEF were around 20% less likely to experience death or hospitalisation over a 4.5-year follow-up period, though this difference was less pronounced in the presence of atrial fibrillation, renal dysfunction, stable angina, or advanced NYHA symptoms [8]. This increase in survival appears to be offset by a decrease in quality of life, as women living with HF have higher self-reported psychological and physical disability scores [10].

The currently available HF guidelines do not address sex-specific recommendations in terms of diagnosis or outcomes, and have minimal content regarding the difference in underlying aetiologies [11, 12]. There is a sex-specific guideline relating to prevention of cardiovascular disease in women [13]; however, it relates primarily to risk factor stratification and treatment optimisation, and is now a decade out of date.

### Imaging for Diagnosis of Heart Failure

HF is a clinical syndrome resulting from structural and/or functional cardiac abnormalities leading to inadequate cardiac output at rest or on exercise or lead to increased intracardiac pressures [14]. Diagnosis is clinical, based on characteristic signs and symptoms; however, confirmation and classification requires quantification of LVEF, which also aids patient management and risk stratification. Echocardiography is generally the first-line imaging modality used for LVEF; however, alternative strategies are available and may have specific advantages in women where acoustic windows may be challenging (Table 1).

**Table 1:** Advantages and disadvantages of different imaging modalities in women with heart failure

	Echocardiography	Cardiovascular computed tomography (CCT)	Cardiovascular magnetic resonance (CMR)	Nuclear (MPS, PET, SPECT)
Advantage (s)	1. High diagnostic accuracy which is increased with 3D 2. Assessment of diastology 3. Gold standard for heart valve assessment 4. Assessment of global longitudinal strain 5. Assessment of ischaemia and viability 6. No ionising radiation 7. Widely available and cost effective	1. Assessment of wall motion, ventricular volumes possible with good correlation to CMR 2. High negative predictive value and overall diagnostic accuracy of coronary artery disease 3. Allows assessment of extracardiac structures	1. Gold standard for EF assessment and ventricular volumes 2. Myocardial tissue characterisation 3. Assessment of ischaemia and viability 4. Allows assessment of extracardiac structures 5. No ionising radiation	1. Good correlation with other techniques for EF assessment. 2. Assessment for ischaemia 3. Assessment for inflammation 4. Good inter- and intraobserver variability.
Disadvantage (s)	1. Acquisition of high-quality images can be challenging due to breast tissue/breast reconstruction 2. Higher inter-observer variability of EF assessment compared to CMR	1. Exposure of breast tissue to ionising radiation 2. Limited temporal resolution 3. Accuracy limited in presence of significant coronary calcium (blooming artifacts) and cardiac arrhythmias	1. High cost 2. Limited availability 3. Presence of arrhythmia limits interpretation	1. Radiation exposure 2. Breast attenuation can result in anterior perfusion defect 3. ECG gated and therefore cardiac arrhythmias limit accuracy. 3. LVEF often overestimated in women due to smaller cavities

*EF* ejection fraction, *CCT* cardiovascular computed tomography, *CMR* cardiovascular magnetic resonance, *MPS* myocardial perfusion scan, *PET* position emission tomography, *SPECT* single-photon emission computed tomography, *ECG* electrocardiogram

### Imaging for Heart Failure Aetiology and Prognosis

**Fig. 1:**
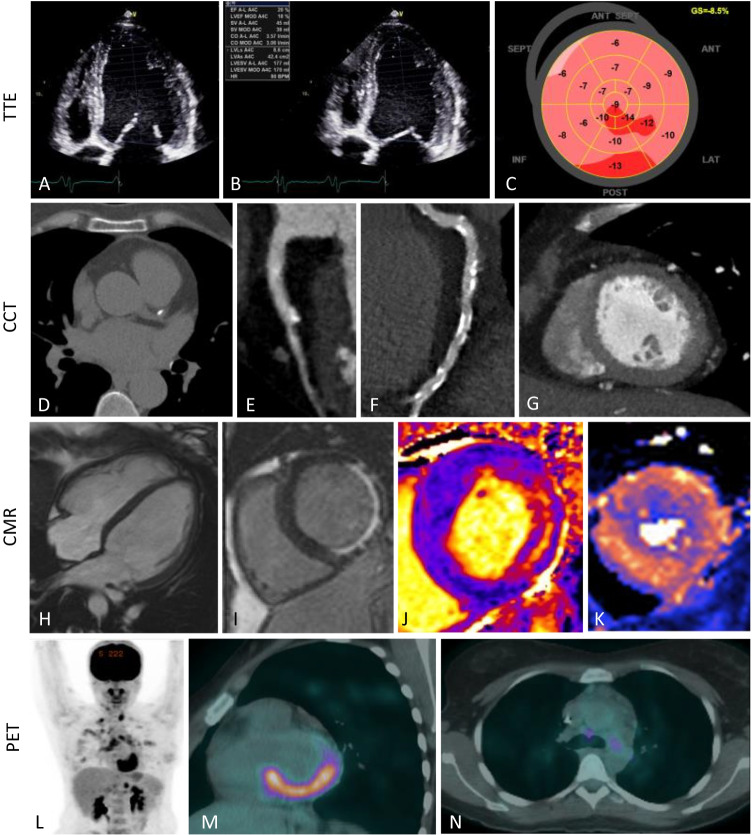
Varying imaging modalities and causes of heart failure in women. TTE, (transthoracic echocardiogram) — images **A–C**. Patient with non-ischaemic cardiomyopathy. Apical 4-chamber view in diastole (**A**) and systole (**B**) showing severely impaired systolic function (LVEF 20%) by Simpson’s Biplane. Strain map showing globally reduced longitudinal strain (**C**). CCT, cardiovascular computed tomography — images D–G. Coronary artery calcium scoring (**D**), curved reformats showing mixed calcified and non-calcified atheroma (**E**) and calcified plaque (**F**), and short axis cine (**G**). CMR, cardiovascular magnetic resonance — images H–K. 4ch cine of dilated heart (**H**), subendocardial infarct on LGE images (**I**), mid-wall inflammation of myocarditis seen on T1 map (**J**), global subendocardial perfusion defect on short axis perfusion map (**K**). PET, positron emission tomography — images **L**–**N**. Focal intense FDG uptake in the left ventricle (**D–E**) and affecting the basal inferoseptum, inferior, inferolateral, and lateral walls (**E**). Axial slice showing FDG avidity in the mediastinal lymph nodes (**F**) in a patient with a history of systemic and cardiac sarcoidosis

Following the diagnosis of heart failure, further imaging may be required to understand the aetiology in order to guide subsequent clinical management. Table 2 summarises the imaging characteristics of the varying imaging modalities discussed in this review and used in different causes of heart failure in women. Figure 1 provides exemplar images of these varying imaging modalities.

**Table 2:** Imaging characteristics of different causes of heart failure in women

	Standard transthoracic echocardiography (TTE)	Advanced echocardiographic modalities	Cardiac computed tomography (CCT)	Cardiac magnetic resonance (CMR) imaging	Nuclear—single-photon emission computed tomography (SPECT), positron emission tomography (PET)
Ischaemic cardiomyopathy	Reduced EF Wall thinning with left ventricular dysfunction—segmental or global	Exercise or dobutamine stress echo to detect regional ischaemia at peak stress Low-dose dobutamine stress testing to assess for myocardial viability Myocardial contrast echo—improved accuracy for identification of obstructive epicardial disease	Identification of anatomical lesion distribution and severity CT-FFR for functional assessment of lesions	Identification of infarction and size via subendocardial-transmural LGE in coronary distribution Viability information from LGE extent—subendocardial versus transmural Stress imaging (e.g., adenosine) to identify inducible perfusion defects	Myocardial perfusion imaging – location and size of focal perfusion defects, reversible or irreversible Viability imaging to detect hibernating myocardium
Microvascular disease (MVD)	Preserved EF +/− LVH	GLS may be abnormal Coronary blood flow by pulse-wave Doppler, rest and stress Myocardial contrast echo—abnormal flow reserve	Confirmation of lack of obstructive CAD	Stress imaging (adenosine) to identify globally reduced myocardial blood flow	PET—quantification of myocardial blood flow, decreased in MVD
Valvular heart disease	Reduced or preserved EF Valvular haemodynamics May be associated with concentric or eccentric LVH, concentric remodelling	GLS may be abnormal Transoesophageal echo for detailed assessment of valvular lesions 3D echo of valve structure for intervention planning	Morphological information of valves and cardiac structures Valve calcification measures Assessment for suitability of structural intervention	Detailed assessment of biventricular size and function, remodelling (concentric/eccentric hypertrophy) Flow assessments for stenotic and regurgitant lesions Myocardial fibrosis assessment using LGE, T1, ECV quantification	
Cancer therapy–related cardiac dysfunction	Reduced EF—LVEF <53% with decrease of >10%	3D-echo LVEF measurements have superior precision GLS decreased by >15%		Precision assessment of LVEF Absence of LGE can help differentiate from other causes of cardiomyopathy	Multi-gated acquisition (MUGA) or blood pool scans historically used for anthracycline toxicity
Peripartum cardiomyopathy	Reduced EF—LVEF decreased to <45%	Contrast echo—identification of LV thrombus		Indicated to rule out other aetiologies—often demonstrates non-specific LGE Identification of LV thrombus	
Autoimmune diseases	Reduced or preserved EF, diastolic dysfunction Pericardial effusion Marantic endocarditis		Aneurysmal lesions Diagnosis of concomitant obstructive CAD	Identification of fibrosis (LGE, T1) and inflammation/oedema (T1, T2) Monitoring of disease activity and response to treatment (T1, T2)	PET can demonstrate increased metabolic activity in the myocardium or vessel wall, also used for monitoring response to treatment
Sarcoidosis	Reduced or preserved EF—global or regional, diastolic dysfunction LV wall thinning, dilatation, aneurysm formation	GLS may be abnormal	Mediastinal lymphadenopathy Peri-lymphatic nodules Osteolytic bone changes	Fibrosis assessment—non-ischaemic pattern LGE (commonly septal, basal, lateral, subendocardial or transmural) Inflammation/oedema (T2)—can identify areas to target for biopsy and monitor disease activity	SPECT can show focal perfusion defects correlating to granulomatous replacement of myocardium PET demonstrates focal FDG uptake in regions of disease activity Non-cardiac uptake may also be seen
Takotsubo cardiomyopathy	Reduced EF—classically akinesis of apical segments with apical ballooning, or mid-ventricular, or reverse variants Identification of LVOT obstruction due to SAM from hyper-dynamic basal contraction		May be used for exclusion of coronary disease	Inflammation in areas of akinesis—T1, T2 Identification of LV thrombus Absence of LGE (generally)	

*EF* ejection fraction, *CT-FFR* computed tomography fractional flow reserve, *LGE* late gadolinium enhancement, *LVH* left ventricular hypertrophy, *GLS* global longitudinal strain, *CAD* coronary artery disease, *MVD* microvascular disease, *LV* left ventricular, *LVOT* left ventricular outflow tract, *SAM* systolic anterior motion of mitral valve

## Coronary Artery Disease

Although non-ischaemic aetiologies predominate in women with HFrEF, CAD remains important and diagnosis is commonly late due to both atypical presenting symptoms and reduced diagnostic accuracy of standard imaging investigations. Women are at increased risk of developing heart failure post-ST-elevation myocardial infarction, and outcomes are worse.

A 2014 consensus statement from the American Heart Association for non-invasive imaging of suspected CAD in women recommended exercise electrocardiograph (ECG) for low-to-intermediate risk women, cardiac computed tomography (CCT) for intermediate risk women, and stress imaging (myocardial perfusion imaging, echocardiography, cardiovascular magnetic resonance (CMR)) for intermediate-to-high risk women [15], although these guidelines were not specific for diagnosing CAD in heart failure. There are, however, different challenges with many of these imaging methods. Exercise ECG is known to be less sensitive and specific in women [16], and nuclear imaging can yield false positive results with perfusion defects in the left ventricular anterior wall due to breast attenuation [17]. Breast irradiation also requires consideration with both nuclear imaging and CCT. The latest European Society of Cardiology heart failure guidelines make no sex-specific recommendations, and propose CCT in patients with low to intermediate risk of CAD and invasive coronary angiography for symptomatic patients with angina despite medical therapy [14]. Advanced imaging has further demonstrated that women have a higher prevalence of non-obstructive CAD than obstructive CAD, despite having more risk factors [18], leading to worse cardiovascular outcomes [19, 20].

## Microvascular Disease

Although epicardial coronary disease is less common, there is growing evidence that coronary microvascular disease or dysfunction (CMD) plays a significant role in the pathophysiology of CAD in women [21]. CMD is defined as impaired vasodilatation of arterioles resulting in a blunted increase in blood flow from rest to stress. CMD appears to be more prevalent in women compared to men [22], and has been shown to be an important prognostic marker linked to increased cardiovascular events in women [21]. It is also thought to play a key role in the pathophysiology of HFpEF, with abnormal coronary flow reserve (CFR) found in HFpEF patients undergoing invasive coronary physiological testing [23] and thought to result in increased myocardial fibrosis [24], driving clinical HFpEF. Further larger studies are required to confirm these findings and to evaluate the prognostic significance in heart failure.

Although conventionally diagnosed via invasive coronary physiological measurements, non-invasive imaging techniques (positron emission tomography [PET] and CMR) are now able to quantify myocardial blood flow (MBF) at rest and with hyperaemia following adenosine or regadenoson, and hence calculate CFR [25, 26]. PET-CT can help to differentiate CMD (reduced CFR and normal epicardial coronary anatomy) from obstructive CAD (reduced CFR and epicardial stenosis). PET is now considered a non-invasive alternative to invasive methods, and the characterisation of CMD by PET has been shown to be clinically prognostic [27, 28]; however, the risk of exposure to ionising radiation should be considered.

Recent technological advances in CMR have permitted automated quantitative measurement of MBF using myocardial perfusion mapping [29] at rest and following adenosine stress, enabling calculation of MBF. The absence of a regional perfusion defect and detection of reduced global stress MBF (<2.25 ml/g/min) has been shown to accurately detect CMD when compared against the standard assessment using invasive measures of index of microcirculatory resistance [30], and may be of benefit in women with HF, particularly with symptoms of chest pain but no epicardial coronary disease.

## Cancer Treatment–Related Cardiac Dysfunction

There is increased recognition of the importance of healthy survivorship in oncology, with prioritisation of early detection and management of treatment-related complications within clinical guidelines [31]. Many cancer therapies including those used to treat breast cancer (anthracyclines and HER-2 therapies) can lead to cardiac complications including HF, hence the introduction of serial surveillance imaging for cancer therapy-related cardiac dysfunction (CTRCD). CTRCD is defined as a decrease in LVEF by 10 percentage points, to a value less than 50% using echocardiography [14, 32]. Sex differences in incidence and mortality are well established across many different cancer types, and many of the malignancies with female preponderance (notably breast) are treated with potentially cardiotoxic treatments making surveillance screening especially important.

Echocardiography is the recommended first line for assessment of cardiotoxicity in all published oncology and cardiology guidelines [31–33•] due to its wide availability, safety profile, lack of ionising radiation, patient tolerability, and cost effectiveness. However, 2D echocardiography depends on good-quality acoustic windows which is commonly challenging, particularly for breast cancer patients following mastectomy or reconstructive implants. Reproducibility of LVEF by 2D echocardiography is around 10% [34], the same threshold for diagnosis of CTRCD, leading to concerns regarding the use of 2D echocardiography for serial surveillance screening. Whilst 3D echocardiography provides superior accuracy and precision due to the lack of geometric assumptions, it may not be feasible in all patients as it too depends on quality acoustic windows [34].

Echocardiography-derived global longitudinal strain (GLS) has been increasingly adopted by guidelines as an adjunctive early biomarker for diagnosis of CTRCD. Recent data from a study of strain-guided management of potentially cardiotoxic chemotherapy [35•] (94% female participants) found that patients following a GLS (as compared to LVEF)–guided pathway for administration of cardioprotective medications had less cardiotoxicity, although LVEF reductions were similar in the two groups. Further data is needed, to more clearly determine the role of GLS in this context.

CMR overcomes the reliance on acoustic windows and is currently recommended for CTRCD surveillance where echocardiographic images are suboptimal or conflicting, or where discontinuation of chemotherapy is considered [32, 35•]. CMR-derived LVEF demonstrates superior reproducibility with a minimum detectable difference of 5.9% [36] and is therefore of significant benefit in such patients. Higher operational costs with more limited availability however preclude it from more widespread use in this context. CMR may also provide additional insights into the underlying mechanisms of cardiotoxicity given its tissue characterisation techniques; late gadolinium enhancement (LGE) imaging and parametric mapping methods (T1, T2, and ECV mapping) for identifying and quantifying myocardial injury and oedema. LGE is not commonly found with CTRCD secondary to anthracyclines and/or trastuzumab [37]; therefore, the absence of LGE could help distinguish anthracycline- and/or trastuzumab-related cardiomyopathy from unrelated cardiomyopathies. CMR with T1, T2, and ECV mapping has shown acutely elevated values in those with acute toxicity, although studies have been small thus far [38].

Of note, multi-gated acquisition scans were historically used first line to monitor for anthracycline toxicity; however, the associated radiation exposure and inability to interrogate the wider cardiovascular structures or measure strain mean that echocardiography should be used in preference [39].

## Peripartum Cardiomyopathy

Peripartum cardiomyopathy (PPCM) is a form of HFrEF which develops either during the last trimester or early within the postpartum period [40•]. PPCM is generally a diagnosis of exclusion but a detailed clinical history and relevant clinical tests are required to rule out other important differential diagnoses of HF in this context. Echocardiography is the first-line imaging modality, with LVEF <45% used for diagnosis [41]. CMR is commonly requested for tissue characterisation where the aetiology remains unclear, and to rule out alternative diagnoses [42]. In one multicentre study involving 34 patients, 71% of patients had a non-specific LGE pattern [43], and whilst there was no typical LGE patterns specific to PPCM, its use can help to determine other differentials such as myocarditis. Both CMR and contrast echocardiography can also be important for assessing the complications of PPCM such as LV thrombus formation, which can occur in 10–17% of cases [44].

## Sarcoidosis

Sarcoidosis is a multi-organ, systematic granulomatous disorder of unknown cause which has a slightly higher prevalence in females [45]. The characteristic features of sarcoidosis on imaging generally include bilateral hilar lymphadenopathy, peri-lymphatic nodules (CT), osteolytic bone changes (CT/MRI), and parotid uptake on nuclear imaging [46].

The prevalence of cardiac sarcoidosis has increased significantly over the past decades, and HF at presentation is noted to carry a particularly poor outcome [47]. Of those with cardiac sarcoidosis, isolated cardiac sarcoidosis has been reported in cases ranging from 27 to 54%, and these patients with isolated cardiac involvement were found to have worse LV systolic function at presentation compared to those with systemic sarcoidosis [48]. Generally, echocardiography is used for suspected sarcoidosis, with commonly described findings including impaired right or left ventricular systolic or diastolic function, regional wall motion abnormalities, aneurysms, focal wall thinning, and impaired GLS [49]. Nuclear imaging using SPECT may identify focal perfusion defects which may correspond to granulomatous replacement of myocardium, and FDG-PET can be useful to detect active cardiac sarcoidosis via increased FDG uptake [49] suggestive of inflammation. Focal perfusion defects on cardiac PET have been shown to correlate with higher risk of cardiac death or ventricular tachycardia [50], which can be helpful prognostically. Finally, CMR has a high sensitivity and specificity for detecting cardiac involvement in sarcoidosis, where scarring may be extensive and detected via LGE imaging, and commonly seen in the basal anteroseptum. Notably, LV dysfunction in sarcoidosis is generally accompanied by scar, and alternative diagnoses should be considered if LV impairment is seen without LGE on CMR. Alongside scar assessment for diagnosis, disease activity can be monitored with CMR via assessment of oedema and inflammation detectable with T2-weighted imaging, with quantification via T1 and T2 parametric mapping. This can be used to identify areas for endomyocardial biopsy which can increase the sensitivity of tissue diagnosis as well as monitoring response to therapy [49].The presence of high burden LGE on CMR in patients cardiac sarcoidosis has additionally been shown to be a marker of poor prognosis with patients being at increased risk of major adverse cardiac events [51•].

## Takotsubo Cardiomyopathy

Takotsubo or stress-induced cardiomyopathy (TTC) has a clear female preponderance with 80–90% of cases found in women [52, 53]. Triggers have more commonly been shown to be emotional in females and physical in males [53, 54]. Initial diagnosis is often via echocardiography, although obstructive CAD generally should be excluded using invasive angiography or urgent CCT if available. The majority of TTC patients display a classical pattern of regional wall motion abnormalities with circumferential hypokinesia/akinesia of the apical LV segments, with normal or hyper-dynamic contraction of the basal segments, giving the appearance of LV apical ballooning. There are also other recognised phenotypes of TTC, including the midventricular-variant characterised by akinesia of the midventricular LV with hyper-dynamic basal and apical contraction, and the reverse variant, which demonstrates basal LV akinesia with hyper-dynamic apical LV contraction, as well as right ventricular involvement with akinesia.

CMR can provide additional information beyond echocardiography in TTC. CMR may demonstrate increases in T2 signal intensity and native T1, T2, and ECV values co-located to the wall motion abnormality [55], which can persist after function normalises. LGE is not typically a feature of TTC; however, recent data has emerged showing that small amounts of LGE may be identified acutely in 10–40% of patients [56]. This LGE is usually less bright (“low-intensity LGE”) compared to the LGE associated with myocardial infarction and myocarditis, and is reversible.

Complications can occur in TTC that can be identified on imaging. These include pleural and pericardial effusions, LV thrombus, and LV outflow tract obstruction with systolic anterior motion of the mitral valve due to the hyper-dynamic basal contraction. Echocardiographic features are similar between the sexes, although an increased rate of LV thrombus has been observed in males [52].

## Autoimmune Diseases

Autoimmune rheumatic diseases (ARDs) have a sex bias towards women of approximately 2:1, and may be higher (7:1 for systemic lupus erythematosus (SLE)) [57]. The risk of incident cardiovascular disease is significantly higher in patients with rheumatoid arthritis [58], SLE [59], and systemic sclerosis [60] and related coronary disease outcomes in these populations are worse [61]. Alongside ischaemia (which is often poorly diagnosed and treated), causes of heart failure in patients with ARDs include myocarditis (with or without myocardial fibrosis), and valvular disease [62•]. Whilst the majority of disease-modifying anti-rheumatic drugs have no effect on major adverse cardiac events [63], some anti-rheumatic drugs have also been demonstrated to have adverse cardiovascular effects, which rarely have been reported to cause restrictive cardiomyopathy during prolonged use (chloroquine) and to precipitate episodes of acute congestive HF (cyclophosphamide) or worsen existing heart failure (TNF-alpha inhibitors) [64, 65].

Echocardiography and CMR are the two most commonly utilised modalities to assess ARD-related heart failure, with CMR favoured for tissue characterisation to assess for active inflammation and guide risk stratification and therapy in the future [66]. Current guidelines on the use of CMR in rheumatology patients with HF note the usual pattern of diastolic dysfunction with a low prevalence of systolic abnormalities seen related to ARDs, and highlight the technique’s ability to make relevant findings relating to fibrosis and inflammation that may relate to ARD activity [67]. In addition to its high diagnostic accuracy for CAD [68], stress CMR provides a functional imaging option that avoids radiation exposure for younger females — particularly those who are unable to exercise to an adequate level due to arthritis or other musculoskeletal limitations.

PET/SPECT can be useful in the diagnosis of ARD-related myocarditis or vasculitis by demonstrating increased metabolic activity in the myocardium or circumferentially in a region of affected vessel walls [69]. PET can be useful for monitoring response to treatment, with metabolic changes being identifiable before any anatomical changes that would be identifiable by CCT or MRI [69].

## Valvular Heart Disease Including Aortic Stenosis

Several recent studies have demonstrated that the pathophysiology and clinical presentation in valvular heart disease may be different between women and men — particularly in aortic stenosis, with increasing recognition of the impact of myocardial remodelling and fibrosis on both symptoms and outcome. For example, despite having a higher LVEF at presentation, women are more likely to have paradoxical low-flow low-gradient aortic stenosis compared to men and this may be contributory to the pathophysiology of aortic stenosis in women and later referrals for intervention, despite paradoxical low-flow low-gradient aortic stenosis carrying a worse prognosis [70].

Early diagnosis is of paramount importance to improve clinical outcomes in women with aortic stenosis, and echocardiography is recommended first line [71] and is gold standard for non-invasive haemodynamic assessment. Recent data has, however, shown added value of other modalities for better phenotyping of patients with aortic stenosis and further interrogating sex differences in the pathophysiology. At a valvular level, women reach a similar haemodynamic degree of stenosis severity with lower levels of valvular calcification compared to men, necessitating different thresholds for determining severe stenosis using multi-detector computed tomography; current suggested thresholds are 1200 Agatston units in women and 2000 Agatston units in men [71, 72]. Explanted stenotic valves show differing fibrosis scores, adding further evidence to sex-related differences in underlying pathophysiology [73]. At a ventricular level, myocardial remodelling patterns with severe aortic stenosis differ between sex based on evidence from CMR that is not apparent using 2-dimensional echocardiography. One study [74] included 168 patients (45% female) undergoing surgical intervention for severe AS, and showed women were significantly more likely to have normal LV geometry or concentric remodelling and increased LVEF compared to men. Men, however, more commonly displayed concentric hypertrophy or eccentric hypertrophy with larger indexed volumes and a maladaptive phenotype which resulted in a lower LVEF, higher cardiac blood biomarkers (NT-proBNP and hsTnT), and more focal and diffuse fibrosis. The relatively lower myocardial fibrosis seen with aortic stenosis in females has also been demonstrated in a multicentre CMR study with LGE imaging, which showed that the presence of LGE was associated with adverse prognosis in both sexes [75•].

## Special Considerations: Radiation and Pregnancy

Cardiovascular disease is an important cause of morbidity and mortality during pregnancy [76], and therefore, cardiovascular assessment is commonly required. There are unique challenges to overcome when imaging in pregnancy including maternal and foetal radiation, exposure to magnetic fields during MR scans, and foetal exposure to contrast agents. Therefore, the risks and benefits must be carefully weighed and non-ionising imaging modalities such as echocardiography and MRI should be used first line where possible.

### Radiation Exposure

Radiation exposure has the potential for both stochastic effects and deterministic effects on the developing foetus and the risks are highest between 3rd and 8th weeks’ gestation [77]. Stochastic effects (such as inducing malignancy) are the result of cellular damage at DNA level and the radiation dose-effect relationship is unpredictable. Deterministic effects, in contrast, are predictable effects once threshold radiation doses have been exceeded and these effects are due to multicellular damage. Theoretical risks depending on timing of radiation exposure include malformations and spontaneous death.

With regard to stochastic effects, an exposure of 50 mGy is considered to double the relative risk of childhood cancer from 0.1 to 0.2% and traditionally a threshold of 150mGy is used for deterministic effects [77]. However, if the benefit outweighs the risk, then an informed discussion between the patient and clinician is required prior to the use of ionising radiation. It is important to note that the dose of radiation to the foetus from cardiac imaging is generally low if the foetus can be kept out of the direct X-ray beam, and shielding used where appropriate. The dose to a foetus from a prospectively gated CCT is usually around 1 mGy, 5–17mGy for radionuclide SPECT, 2 mGy for PET, and 0.074 mGy for an invasive coronary angiogram [78]. Iodinated contrast agents are known to cross the human placenta; however, teratogenic effects have not been detected clinically after the administration of these media, despite a theoretical potential to induce foetal hypothyroidism. The American College of Radiology therefore recommends that iodinated contrast should therefore not be withheld if indicated during pregnancy. Foetal radiation exposure may be higher with nuclear imaging due to the distribution of radiopharmaceuticals which may concentrate in the maternal bladder with proximity to the placenta. Despite this, doses from typical diagnostic nuclear medicine and PET agents are not expected to approach exposures exceeding 50 mGy [78]. Doses should be kept as low as possible, and the mother should be encouraged to keep hydrated in order to encourage frequent voiding.

Radiation exposure to breast tissue is also of concern. It has been predicted that a CCT could result in a lifetime excess relative risk for breast cancer of 1.4–2.6% and 0.2–0.4% in women aged 25 and 55 years respectively [79]. It is also important to note that lactating breast is more radiosensitive than in the non-pregnant state, and the principles of “as low as reasonably achievable” should be stringently applied. Radiation reduction techniques including cranial breast displacement have been shown to reduce the breast skin entrance dose during CCT [80].

### MRI

There has been no evidence to date to suggest that MRI (up to 3T) causes harm to the baby in utero [81]; therefore, CMR can be performed safely in pregnancy, and both the American College of Radiology and the European Society of Cardiology recommend that diagnosis of complex cardiac disease should use MRI where other basic modalities (principally echocardiography) are inadequate. It is however generally recommended to wait until after 12 weeks gestation where possible and to scan at the lowest possible field strength. However, the use of gadolinium contrast agents has been associated with infiltrative skin conditions, rheumatological conditions, and an increased risk of stillbirth or neonatal death, and hence is best avoided [81]. In the post-partum period, there is no evidence to suggest harm to the baby from gadolinium during breastfeeding [82]. The yield from CMR in pregnancy is high — in the largest series of its kind, Herrey et al. showed in data from 84 patients that CMR changed management in 35% and in 50% of patients who received contrast, of whom almost half were undergoing scans for cardiomyopathy/myocarditis [83•].

## Conclusions

Diagnosis and stratification of HF is generally performed first line using transthoracic echocardiography. Understanding the aetiology of heart failure is central to ongoing management, with non-ischaemic causes more commonly found in women. This generally involves use of one or more advanced imaging techniques including CMR or PET for tissue characterisation, and CCT or nuclear myocardial perfusion imaging for coronary assessment. There are additional specific considerations for imaging in women including radiation risks and challenges with imaging during pregnancy. There is now a clear unmet need for cardiology and imaging societies to provide imaging specific guidelines for women with heart failure.
